# FRS2α Regulates Erk Levels to Control a Self-Renewal Target Hes1 and Proliferation of FGF-Responsive Neural Stem/Progenitor Cells

**DOI:** 10.1002/stem.488

**Published:** 2010-07-22

**Authors:** Takuya Sato, Takuya Shimazaki, Hayato Naka, Shin-Ichi Fukami, Yasushi Satoh, Hideyuki Okano, Irit Lax, Joseph Schlessinger, Noriko Gotoh

**Affiliations:** a Division of Systems Biomedical Technology, Institute of Medical Science, The University of TokyoTokyo, Japan; b Department of Physiology, Keio University School of MedicineTokyo, Japan; c Department of Anesthesiology, National Defense Medical CollegeSaitama, Japan; d Department of Pharmacology, Yale University School of MedicineConnecticut, USA

**Keywords:** MAPK, Neural stem cells, Neural progenitor cells, Signal transduction, Self-renewal, FRS2, SNT1, Grb2, Shp2

## Abstract

Fibroblast growth factor (FGF) is among the most common growth factors used in cultures to maintain self-renewal and proliferative capabilities of a variety of stem cells, including neural stem cells (NSCs). However, the molecular mechanisms underlying the control by FGF have remained elusive. Studies on mutant mice of FGF receptor substrate 2α (FRS2α), a central mediator for FGF signaling, combined with FRS2α knockdown or gain-of-function experiments, allowed us to dissect the role of FGF signaling for the self-renewal and proliferation of NSCs and to provide novel molecular mechanisms for them. We identified Hes1 as a novel self-renewal target of FGF-signaling. Quantitatively different levels of Erk activation mediated by FRS2α may regulate self-renewal of NSCs and proliferation of neural stem/progenitor cells (NSPCs); low levels of Erk activation are sufficient for the former, however, higher levels are required for maximum activity of the latter. Thus, FRS2α fine-tunes the FGF-signaling to control qualitatively different biological activities, self-renewal at least partly through Hes1 versus proliferation of NSPCs. Stem Cells 2010; 28:1661–1673.

## INTRODUCTION

Fibroblast growth factor (FGF) signaling mediates diverse cellular responses during embryonic development and plays critical roles in the physiological and pathological processes of the adult organism [1]. Particularly, FGF signaling plays a crucial role in the self-renewal and proliferation of various stem cells such as human embryonic stem cells, human-induced pluripotent stem cells, trophoblast stem cells, and neural stem cells (NSCs) [2,3]. Moreover, it has been recently reported that cancer stem cells derived from various types of human tissues, such as brain and breast tissues, can be cultured in vitro in the presence of FGF [3]. Many studies indicate that FGF signaling has critical functions in both self-renewal and proliferation of neural stem/progenitor cells (NSPCs) in vitro and in vivo [4–7]. The molecular mechanisms by which FGF controls the two distinct processes, that is, the self-renewal and proliferation of stem cells, are still unclear. The lack of such knowledge prevents the development of appropriate methods to cultivate stem cells with suitable qualities, which are critical to regenerative medicine such as cell replacement therapy for disease-damaged tissues or organs.

The membrane-linked docking/scaffolding adaptor FGF receptor substrate 2 (FRS2)-α acts as a central mediator for FGF signaling [1,8-11]. Once stimulated by FGF, FRS2α becomes tyrosine-phosphorylated and creates two specific binding sites for Shp2, an SH2 domain-containing tyrosine phosphatase and four binding sites for the adaptor protein, Grb2, leading to activation of Ras-Erk pathway [12,13]. We previously examined contribution of each site for FGF-induced activation of Erk by exogenously expressing wild-type FRS2α or its mutants in *Frs2*α^−/−^ mouse embryonic fibroblasts [13]. Strong activation of Erk is observed in cells expressing the wild-type FRS2α or FRS2α-4F, in which tyrosine residues of Grb2-binding sites are replaced with phenylalanine, though the cells expressing FRS2α-4F shows slightly lower levels of Erk activity than wild-type. FRS2α-2F, in which tyrosine residues of Shp2-binding sites are replaced with phenylalanine, shows more reduced levels of activation of Erk and FRS2α-6F, a combined mutant of both Grb2- and Shp2-binding sites, shows least levels of Erk activity. Thus, the Shp2-binding sites in FRS2α have a primary role in the activation of Erk, whereas the Grb2-binding sites have a secondary role in the activation of Erk [13–15].

*Frs2*α is ubiquitously expressed during development [16]. To analyze the functions of FRS2α, we previously constructed a *Frs2*α-knockout mouse and two knockin mice that express a mutant form of FRS2α whose tyrosine residues of Grb2 (*Frs2a^4F^* mutant)- or Shp2 (*Frs2a^2F^* mutant)-binding sites are replaced with phenylalanine and demonstrated its critical functions in multiple developmental processes that are dependent on FGF-signaling. Embryos of the *Frs2a*-knockout mouse have defects in the FGF4-dependent maintenance of trophoblast stem cells and shows developmental retardation, resulting in embryonic lethality by E8 [12]. *Frs2a^4F/4F^* mice can survive as adults and show no gross morphological defects except for eyelid developmental defects that arise with low penetrance [15]. *Frs2a^2F/2F^* mice display multiple developmental defects along with perinatal death. Embryos of *Frs2a^2F/2F^* mice lack carotid bodies [17], have defective eye development and show anophthalmia or microphthalmia [15].

The expression of *Frs2*α is particularly strong in the ventricular zone (VZ) of the developing cortex, where radial glial cells (embryonic NSPCs) are localized [16]. In addition, *Fgf*s, together with the genes for their receptors (*Fgfr*s), are also expressed in the VZ [18–21]. By analyzing the *Frs2α^2F^* mutant mice, we previously demonstrated that these sites are required for the proliferation of neural progenitor cells but are dispensable for the self-renewal of NSCs both in vivo and in vitro [22]. However, previous studies indicate that FGF signaling is required for the self-renewal of NSCs [23,24]. Given that FRS2α is a central mediator of FGF signaling, we decided to further explore the function of FRS2α in NSCs.

In this study, we performed gain-of- and loss of-function analysis of FRS2α both in vitro and in vivo. These analyses showed that the quantitative regulation of Erk activation levels by FRS2α is important not only for the proliferation of NSPCs but also for the self-renewal of NSCs. Moreover, we identified Hes1 as a novel target of FGF-signaling for the self-renewal of NSCs. Our results suggest that FRS2α stands as a central player in the regulation of the delicate balance between the numbers of NSCs and progenitor cells to maintain proper homeostatic number of neural cells in cultural systems and cortical development.

## MATERIALS AND METHODS

### Cell Culture

Primary neurospheres were established from the telencephalons of E12.5 or E14.5 embryos. Telencephalons were dissociated by trituration. Cells were then cultured in basal serum-free medium [22] containing 20 ng/ml FGF2 (PEPROTECH, Rocky Hill/NJ, http://www.peprotech.com/) or 20 ng/ml epidermal growth factor (EGF; TOYOBO, Osaka/Japan, http://www.toyobo.co.jp/e/) for 4–5 days. The resulting primary neurospheres were dissociated, either infected with virus or not, and then cultured in the presence of the same growth factor as the primary neurospheres for 7–9 days. The resulting secondary neurospheres were dissociated, and Green Fluorescent Protein (GFP)- or ZsGreen-positive (virus infected) cells were sorted by Fluorescence Activated Cell Sorting (FACS) (Beckton Dickinson FACSAria) and plated into 96-well plates (Corning, Corning/NY, http://www.corning.com/index.aspx). The cells were cultured again in the presence of the same growth factor as the secondary neurospheres, unless noted, for 9–12 days. Next, the size and number of tertiary neurospheres were determined. The Mek inhibitors U0126 (Calbiochem, Darmstadt/Germany, http://www.merck-chemicals.jp/life-science-research/calbiochem/japanese/c_PmSb.s1ON3EAAAEj0uBXhFCU), PD98059 (Cell Signaling), and γ-secretase inhibitor DAPT (*N*-[*N*-(3,5-difluorophenacetyl)-l-alanyl]-*S*-phenylglycine *t*-butyl ester) (Calbiochem) were dissolved in dimethyl sulfoxide (DMSO) and added to the culture medium at indicated concentrations. Half of the culture medium with inhibitors was changed every day. For adherent culture of NSPCs, cells were seeded to a culture dish precoated with 15 μg/ml poly-(l-ornithine) (Sigma) and 1 μg/ml fibronectin (Sigma, St. Louis, MO, http://www.sigmaaldrich.com/united-states.html).

### Statistical Analysis

Data in the graphs are represented as mean ± SD. The statistical significance was assessed with Student's *t*-test or Welch's *t*-test according to the variance between the two samples. Statistically significant differences are indicated with an asterisk (*p* < .05) or double asterisks (*p* < .01) in the figures.

## RESULTS

### Overexpression of FRS2α Promotes FGF2-Induced Proliferation and Self-Renewal of NSPCs In Vitro

It has been shown that NSPCs located in the telencephalon can be stimulated to proliferate in response to FGF2 or EGF stimulation and form spherical cell clusters called neurospheres in vitro [25]. The neurosphere cells are composed of heterogenous cell populations, including stem cells and multipotent progenitor cells [26]. Because self-renewing NSCs are capable of reforming new neurospheres and each neurosphere is derived from a single NSC, the frequency of neurosphere formation corresponds to the self-renewing capacity of the NSCs in the original neurospheres [22,27,28].

We first performed a gain-of-function analysis of FRS2α in vitro. We used wild-type FRS2α and two mutants: FRS2α-6F and FRS2α-8V. FRS2α-6F has all six tyrosine phosphorylation sites replaced by phenylalanine residues. FRS2α-6F does not interact with Shp2 or Grb2, and the introduction of an FRS2α-6F-expressing vector into FRS2α-deficient mouse embryonic fibroblasts could not rescue the activation of Erk in these cells in response to FGF [13,14]. The other FRS2α mutant FRS2α-8V has eight threonine phosphorylation sites replaced by valine residues, and it is thought to act as a dominant active form of FRS2α, which is sensitized to FGF stimulation due to the lack of negative regulatory phosphorylation sites [29].

We obtained NSPCs from the telencephalons of embryonic day (E) 14.5 mouse embryos and cultured them in the presence of FGF2 or EGF to obtain primary neurospheres. The primary neurospheres were dissociated to single cells and transduced with retroviral expression vectors encoding wild-type FRS2α or its mutant forms, FRS2α-6F or FRS2α-8V, together with GFP and the cells were cultured in the presence of FGF2 or EGF to form secondary neurospheres. The secondary neurospheres were dissociated, and cells expressing GFP were sorted by FACS; these sorted cells were cultured again in the presence of FGF2 or EGF to form tertiary neurospheres.

**Figure 1:**
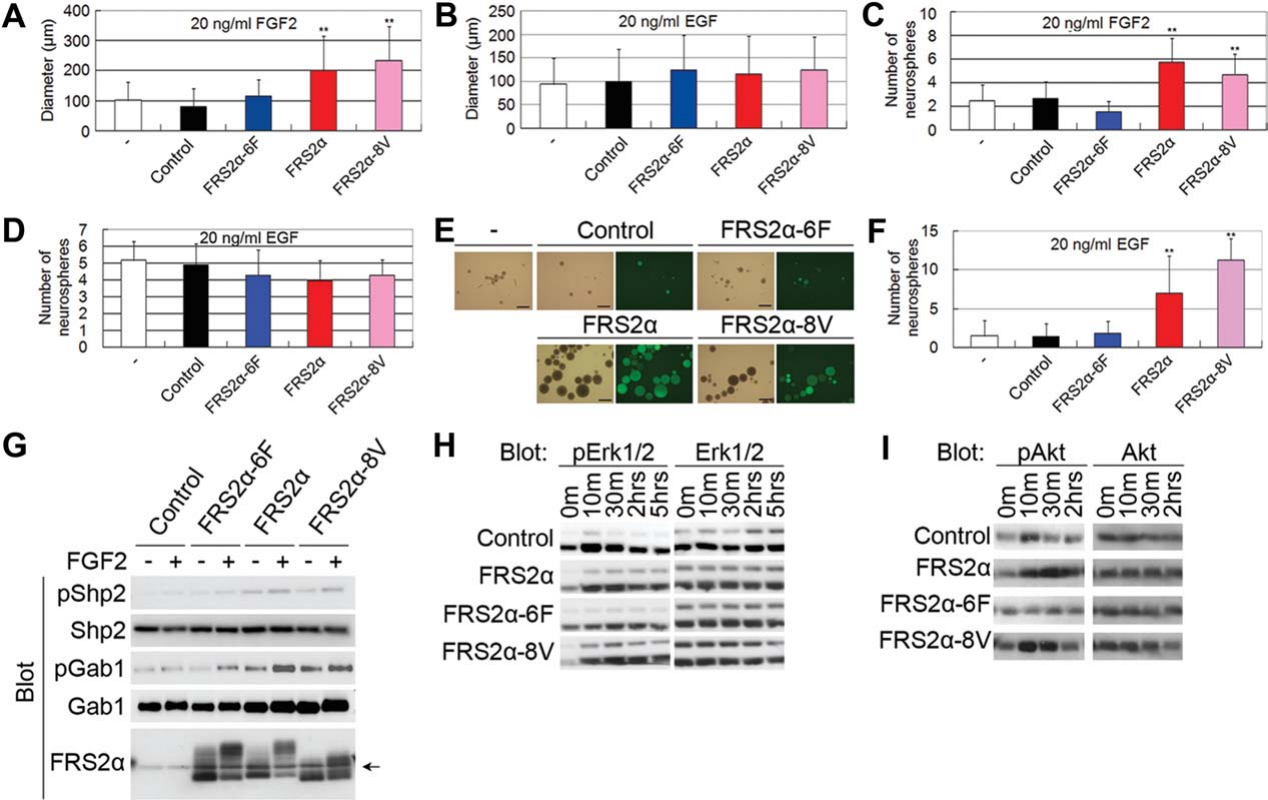
Overexpression of FRS2α promotes FGF2-induced proliferation and self-renewal of neural stem/progenitor cells (NSPCs) in vitro. **(A–E):** NSPCs were infected with retrovirus and cultured in the presence of FGF2 **(A, C)** or EGF **(B, D)**. Then, the diameter (**A, B)** or number (**C, D)** of resulting neurospheres was determined. A minus (−) sign indicates that the virus was not infected. Scale bars = 500 μm **(E)**. **(F):** EGF-induced tertiary neurospheres were produced from FGF2-induced secondary neurospheres, and the number of neurospheres was determined. Experiments were performed at least two times with similar results. **(G–I):** Neurospheres were starved and then stimulated with FGF2. Then, the lysates were subjected to immunoblotting. The arrow in **(G)** indicates nonspecific bands. Abbreviations: EGF, epidermal growth factor; FGF, fibroblast growth factor; FRS2α, FGF receptor substrate 2α.

When NSPCs were cultured in the presence of FGF2, cells expressing FRS2α or FRS2α-8V, but not FRS2α-6F, formed significantly larger tertiary neurospheres than did control neurospheres transduced with an empty vector (Fig. 1A). In contrast, when NSPCs were cultured in the presence of EGF, the expression of FRS2α or its mutant forms had no effect on the size of neurospheres (Fig. 1B). Interestingly, the expression of wild-type FRS2α or FRS2α-8V, but not FRS2α-6F, also significantly increased the frequency of FGF2-induced tertiary neurosphere formation (Fig. 1C) but not that of EGF-induced neurosphere formation (Fig. 1D). We obtained similar results using NSPCs from E12.5 telencephalons (Fig. 1E and Supporting Information Fig. 1). These results suggest that the FGF2-induced tyrosine phosphorylation of FRS2α is important not only for the proliferation of NSPCs but also for the self-renewal of NSCs in vitro. To confirm this further, we passaged FGF2-induced secondary neurospheres and then cultured the NSPCs in the presence of EGF. The frequency of EGF-induced tertiary neurosphere formation was increased when using FRS2α or FRS2α-8V, but not FRS2α-6F, was expressed (Fig. 1F). Expression of FRS2α or FRS2α-8V had no effect on the formation of EGF-induced tertiary neurospheres when EGF-induced secondary neurospheres were used (Fig. 1D). This result indicates that the expression of FRS2α or FRS2α-8V enriched the number of self-renewing NSCs in each neurosphere induced by FGF2 but not by EGF.

To confirm the expression of FRS2α protein in neurosphere cells, we performed western blotting. Strong expression of FRS2α was observed in cells expressing the wild-type or mutant forms (Fig. 1G). Both wild-type FRS2α and FRS2α-6F showed a strong shift in electrophoretic mobility in response to FGF2 (Fig. 1G). Consistent with the notion that the shift is largely caused by the phosphorylation of threonines in FRS2α by activated Erk, FRS2α-8V showed only a slight shift [29]. Western blotting was used to examine the activation of components of the FGF signaling pathways downstream of FRS2α in NSPCs expressing wild-type or mutant forms of FRS2α in response to FGF2. Stronger activation of Erk or Akt was observed in cells overexpressing FRS2α or FRS2α-8V after stimulation with FGF2 compared with FRS2α-6F-expressing cells or control cells (Fig. 1H, 1I). Enhanced Akt activation is consistent with a previous report that Grb2-binding to Gab1 contributes to activation of Akt [13]. We also evaluated the activation of Shp2 and Gab1 by immunoblotting with anti-phospho-Shp2 and anti-phospho-Gab1 antibodies, respectively. Activation of Shp2 and Gab1 was enhanced in cells expressing wild-type FRS2α or FRS2α-8V compared with control cells or cells expressing 6F (Fig. 1G).

### Overexpression of FRS2α Promotes FGF2-Induced Proliferation and Self-Renewal of NSPCs In Vivo

**Figure 2:**
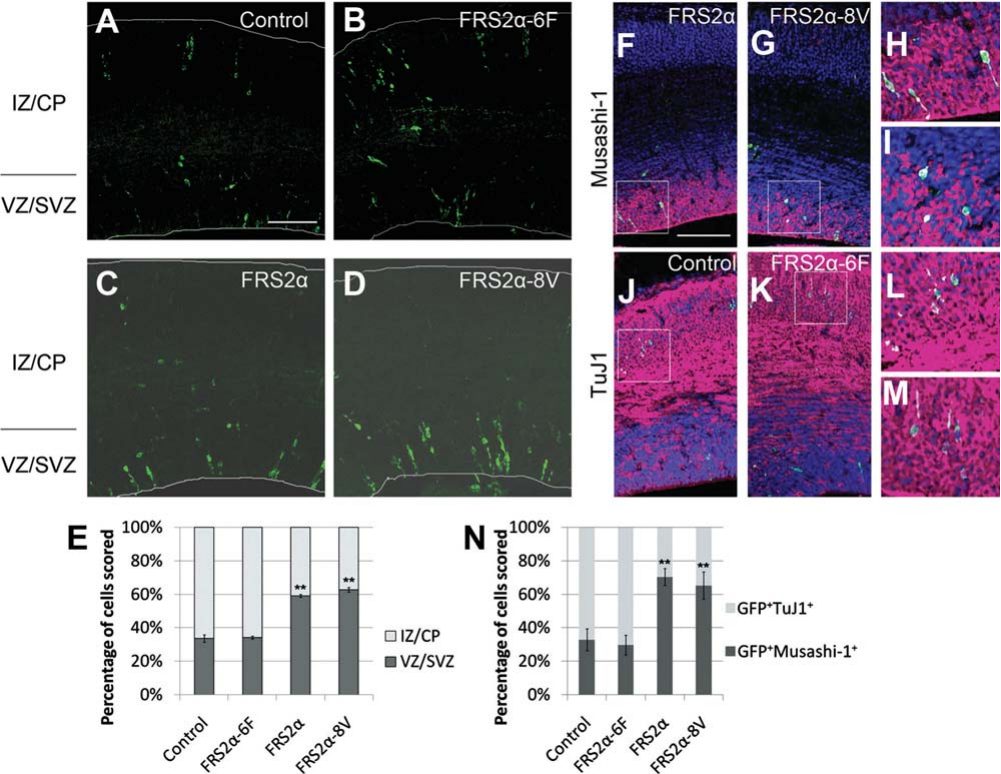
Overexpression of FRS2α represses the differentiation of neural stem/progenitor cells (NSPCs) in the developing cortex. **(A–D):** Retroviruses were injected in utero into the telencephalic ventricles at E12.5. The embryos were then sacrificed at E15.5, and cortical sections were immunostained against GFP (green). **(E):** Quantification of the data presented in **(A–D)**. In each condition, at least 183 cells were counted in 10 randomly selected areas. **(F–M):** Cortical sections of embryo infected with viruses were double-immunostained against GFP (green) and Musashi-1 (magenta) **(F–I)**, or TuJ1 (magenta) **(J–M)**. Nuclear staining is shown in blue. The panels **(H)**, **(I)**, **(L)**, and **(M)** are higher magnifications of the regions indicated by rectangles in **(F)**, **(G)**, **(J)**, and **(K)**, respectively. **(N):** Quantification of the data presented in **(F–M)**. In each condition, at least 64 cells were counted in 10 randomly selected areas. Scale bars = 50 μm. Abbreviations: CP, cortical plate; GFP, Green Fluorescent Protein; FRS2α, FGF receptor substrate 2α; IZ, intermediate zone; SVZ, subventricular zone; VZ, ventricular zone.

To examine the function of FRS2α in the self-renewal of NSCs in vivo, we overexpressed wild-type FRS2α or its mutant forms in the developing mouse cortex using a retroviral expression system. We injected retrovirus into the telencephalic lateral ventricles of E12.5 mouse embryos in utero and then sacrificed them at E15.5. At these stages, NSPCs are normally localized at the VZ/subventricular zone (SVZ) and generate neurons, and those neurons migrate out of the VZ/SVZ through the intermediate zone (IZ) to settle at the cortical plate (CP) [30]. Immunohistochemistry of the viral GFP marker in cortical sections showed that a large number of control or FRS2α-6F-expressing cells migrate out of the VZ/SVZ into the IZ/CP (Fig. 2A, 2B, 2E). In contrast, many FRS2α- or FRS2α-8V-expressing cells remained in the VZ/SVZ, and the number of cells in the IZ/CP was decreased compared with controls (Fig. 2C–2E). These results imply that the expression of FRS2α or FRS2α-8V, but not FRS2α-6F, represses the differentiation of NSPCs.

To examine the identity of virus-infected cells, we performed double-immunostaining against GFP and TuJ1 (a neuronal marker) or Musashi-1 (a NSPC marker). This analysis showed that the overexpression of FRS2α or FRS2α-8V, but not FRS2α-6F, increased the ratio of NSPCs to neurons (Fig. 2F–2N).

**Figure 3:**
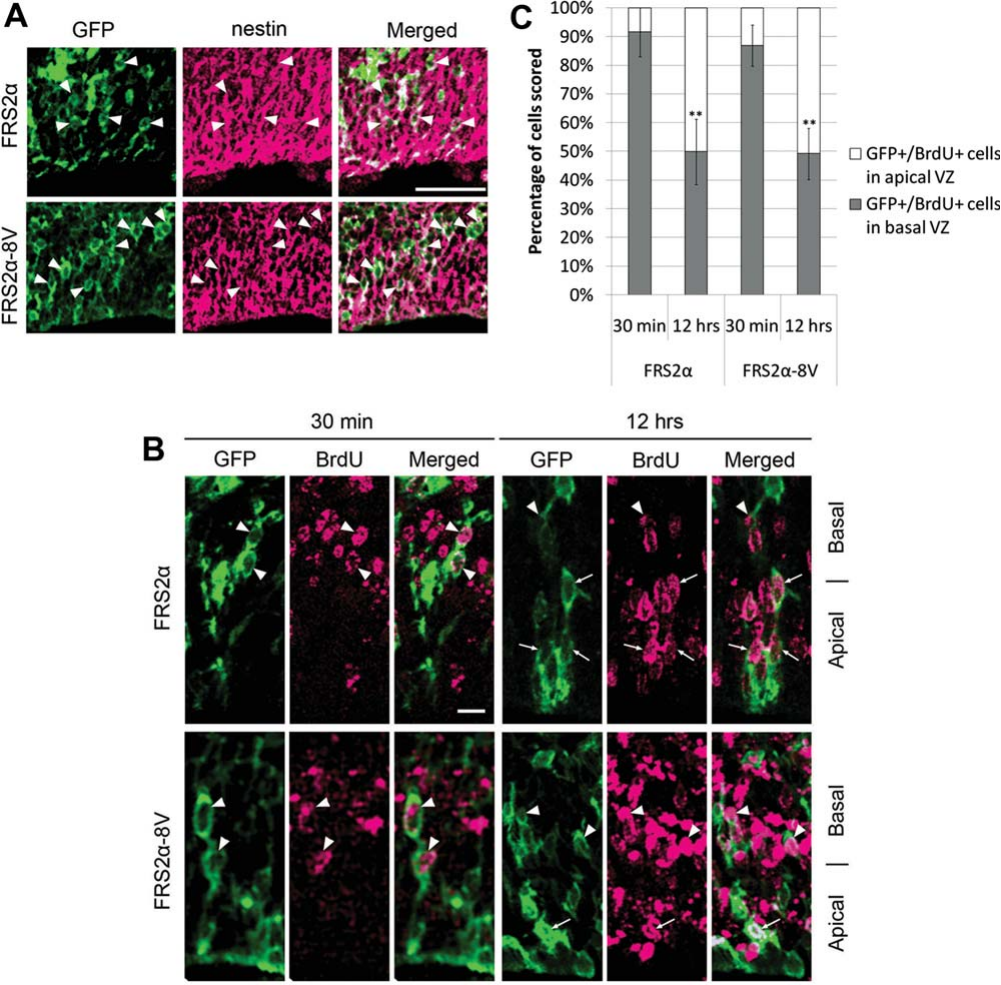
Cells expressing FRS2α or FRS2α-8V show radial glial properties. **(A):** Most GFP-positive cells (green), which express FRS2α or FRS2α-8V, also expressed nestin (magenta), as indicated by the arrowheads. Scale bar = 50 μm. **(B):** The cell bodies of most GFP-positive cells in the S-phase (30 minutes after BrdU administration), identified by BrdU signals (magenta), were located at the basal half of VZ (arrowhead). In contrast, the cell bodies of GFP-positive cells in the G1 phase (12 hours after BrdU administration) were present at both the basal and apical (arrow) halves of VZ. Scale bar = 10 μm. **(C):** Quantification of the data presented in **(B)**. About 300 cells were counted in each condition in 20–32 randomly selected areas. Abbreviations: BrdU, Bromodeoxyuridine; GFP, Green Fluorescent Protein; FRS2α, FGF receptor substrate 2α; VZ, ventricular zone.

To confirm that cells expressing FRS2α or FRS2α-8V are “bona fide” NSCs, we examined whether they express nestin, another marker of NSCs, and undergo interkinetic nuclear migration (INM), a cell cycle-dependent somal translocation process unique to radial glial cells [31]. During the S-phase, the soma of radial glial cells is located at the basal (abventricular) side of VZ, but descends toward the apical (ventricular) surface during the G2 phase. The cell body is present at the ventricular surface during the M phase, and ascends basally during the G1 phase. We coinjected vectors expressing FRS2α or FRS2α-8V together with GFP into E13.5 cortex and performed in utero electroporation, and then sacrificed the embryos at E15.5. BrdU was administered 30 minutes or 12 hours before sacrifice to label cells in the S-phase or mainly the G1 phase, respectively [32]. Then, cortical sections of the embryos were subjected to double-immunostaining with antibodies against GFP and nestin or BrdU. This analysis showed that most cells in the VZ expressing FRS2α or FRS2α-8V were nestin-positive (95.7% ± 5.4% of cells expressing FRS2α among a total of 691 cells counted in 22 randomly selected areas; 97.9% ± 5.5% of cells expressing FRS2α-8V among a total of 717 cells counted in 26 randomly selected areas; Fig. 3A). Although cell bodies expressing FRS2α or FRS2α-8V were present at the basal side of VZ when BrdU was administered 30 minutes before sacrifice, many of them were located at the apical side when BrdU was administered 12 hours before sacrifice (Fig. 3B and 3C). These results strongly suggest that cells expressing FRS2α or FRS2α-8V underwent INM and remained bona fide NSCs.

Together, our results raise the possibility that FGF signaling mediated by tyrosine phosphorylation of FRS2α represses NSPC differentiation and thereby promotes the self-renewal of NSPCs in vivo.

### Grb2-binding sites of FRS2α Are Required for the Maximum Proliferation of NSPCs, But Are Dispensable for the Self-Renewal of NSCs

**Figure 4:**
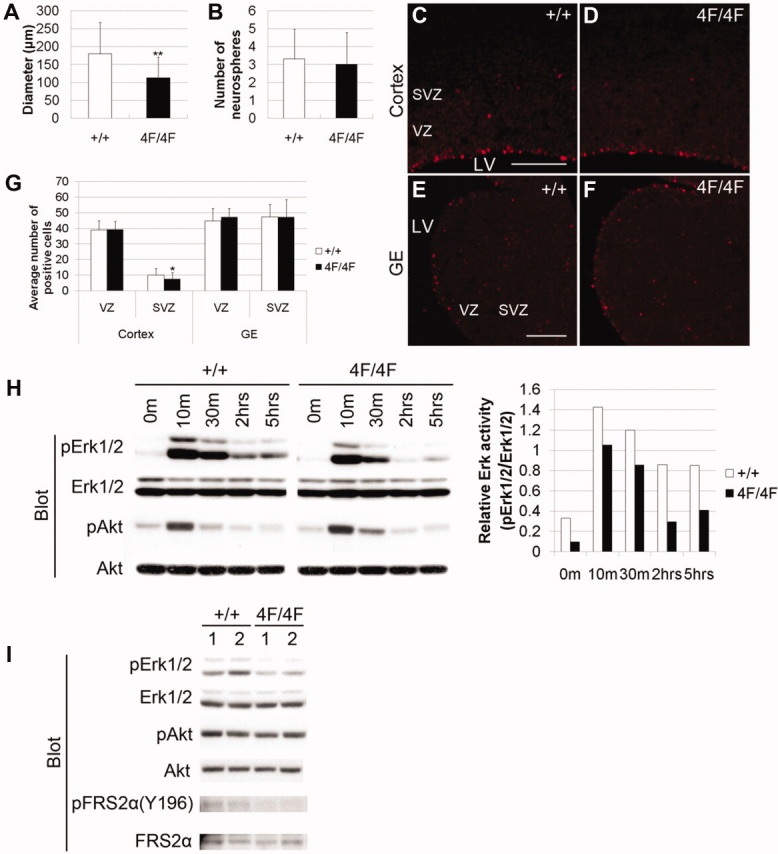
The Grb2-binding sites of FRS2α are required for the maximum levels of the proliferation of neural stem/progenitor cells, but are dispensable for the self-renewal of neural stem cells. **(A, B):** The diameter of the fibroblast growth factor 2 (FGF2)-induced secondary neurospheres derived from E14.5 telencephalon was slightly decreased in the mutant **(A)**. The number of the secondary neurospheres was not significantly different **(B)**. Experiments were performed three times with similar results. **(C–F):** The cortex or GE of E14.5 embryos was immunostained with anti-pH3 antibody (red). Scale bar = 50 μm. **(G)** Quantification of the data presented in **(C–F)**. In each condition, at least 280 cells positive for phospho-histone H3 in VZ or SVZ were counted in 36 randomly selected areas. **(H):** Neurospheres were starved and then stimulated with FGF2. The signal intensities of pErk1/2 and Erk1/2 were quantified, and represented as the relative activity of pErk against Erk1/2 (right panel). **(I):** Brains of E11.5 embryos were lysed and subjected to immunoblotting. Two independent samples are indicated for each genotype. Abbreviations: GE, ganglionic eminence; FRS2α, FGF receptor substrate 2α; LV, lateral ventricle; SVZ, subventricular zone; VZ, ventricular zone.

We have previously demonstrated that the Shp2-binding sites of FRS2α are dispensable for the self-renewal of NSCs [22]; therefore, we next analyzed the function of the Grb2-binding sites of FRS2α using an *Frs2α^4F^* mutant mouse in which phenylalanine replaces four tyrosine residues in the Grb2-binding sites. Although FGF2-induced secondary neurospheres from the telencephalons of E14.5 *Frs2α^4F^* mutant embryos were smaller than those of wild-type embryos (Fig. 4A), the frequency of secondary neurosphere formation was not significantly different (Fig. 4B). Thus, the self-renewal ability of NSCs from *Frs2α^4F/4F^* mutant mice appears to be normal, although the proliferation of mutant NSPCs was slightly decreased in vitro.

Immunohistochemical analysis of the telencephalons of E14.5 embryos using anti-phospho-histone H3 (pH3) antibody, an M-phase marker, showed that the mitotic activity of the mutant telencephalons was normal in the VZ, where radial glial cells divide. Cell division in the E14.5 SVZ of the cortex but not of the ganglionic eminence, where intermediate progenitor cells divide [22,33,34], was slightly reduced (Fig. 4C–4G). Intermediate progenitor cells are derived from radial glial cells and are committed to neuronal cells. This result confirms that the self-renewal ability of NSCs from *Frs2α^4F/4F^* mutant mice appears to be normal, although the proliferation of mutant NSPCs was slightly decreased.

In response to FGF2 stimulation, mutant NSPCs showed moderately decreased Erk activation compared with wild-type cells (Fig. 4H). However, the activation of Akt was not affected in the mutant NSPCs. In addition, western blotting using lysates of E11.5 brain tissue showed that Erk activation, but not the Akt activation level, was moderately decreased in mutant brains (Fig. 4I). Tyrosine phosphorylation of one of the Grb2-binding sites of FRS2α, tyrosine-196, was reduced in mutant brains, confirming that the mutant lacks these sites.

Thus, although the proliferation and Erk activation of *Frs2α^4F^* NSPCs are moderately impaired, the self-renewal ability of the mutant NSCs appears to be normal both in vivo and in vitro.

### Erk Activation via FRS2α Is Required for the Full Activity of FGF-Induced Self-Renewal of NSCs

**Figure 5:**
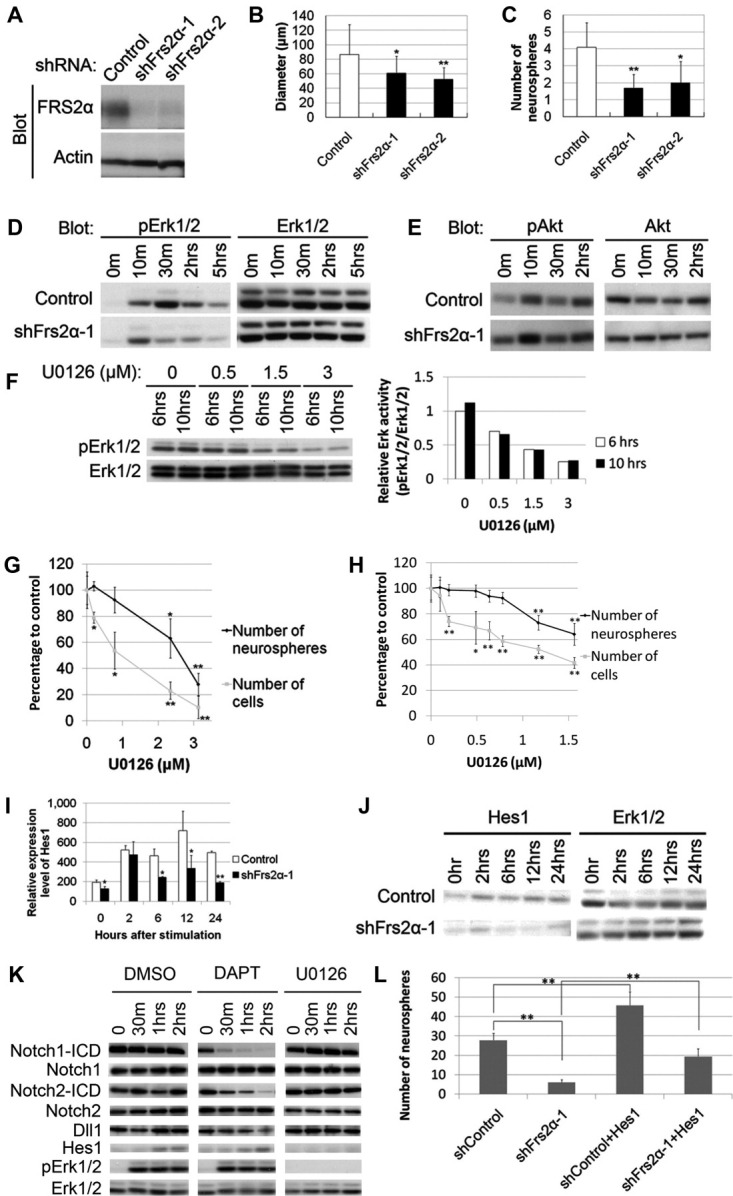
(Overleaf) Erk activation via FRS2α is required for the FGF-induced self-renewal of neural stem cells. **(A):** NIH3T3 cells were infected with retroviral vectors encoding shRNAs. The cells were then lysed and the lysates were subjected to immunoblotting. **(B, C):** Fibroblast growth factor 2 (FGF2)-induced secondary neurospheres infected with retrovirus encoding shRNAs were dissociated, and the cells were cultured again in the presence of FGF2, and the diameter **(B)** and number **(C)** of the resulting tertiary neurospheres were determined. Experiments were performed four times with similar results. **(D, E):** Neural stem/progenitor cells (NSPCs) infected with lentivirus encoding shRNAs were starved and then stimulated with FGF2. Then cells were lysed, and the lysates were subjected to immunoblotting. **(F):** NSPCs were cultured in the presence of FGF2 together with different doses of U0126. Then, the cells were lysed 6 or 10 hours after the initiation of culture. The relative activity of pErk against Erk1/2 is shown in the right panel. **(G, H):** NSPCs were cultured in the presence of FGF2 together with U0126 to form neurospheres. The resulting primary neurospheres were dissociated to single cells and the cell number was counted. Then, NSPCs were cultured again to form secondary neurospheres, and the number of resulting neurospheres was determined. Data are represented as average with SD of at least three separate experiments, and the *y*-axis indicates percentage to the control (0 μM). We counted at least 200 spheres in each experiment. **(I, J):** NSPCs infected with lentivirus were starved and stimulated with FGF2. RNA was extracted from NSPCs for quantitative reverse transcription polymerase chain reaction **(I)** or NSPCs were lysed for immunoblotting **(J)** at each time point. **(K):** NSPCs were starved and pretreated for 1 hour with DMSO (vehicle), 25 μM U0126, or 10 μM DAPT before stimulation with FGF2. Then, cells were lysed for immunoblotting. **(L):** NSPCs were infected with lentivirus expressing shRNAs and retrovirus expressing Hes1 or its empty vector, and cultured in the presence of FGF2. Then, cells were passed and cultured again in the presence of FGF2, and the number of the resulting neurospheres were determined. Experiments were performed three times with similar results. Abbreviations: DAPT, ((N-[N-(3,5-difluorophenacetyl)-L-alanyl]-S-phenylglycine t-butyl ester); DMSO, dimethyl sulfoxide; FRS2α, FGF receptor substrate 2α.

It seems paradoxical that the data from the gain-of-function experiments suggest that tyrosine phosphorylation of FRS2α enhances the self-renewal of NSCs; however, the loss of the Shp2-binding sites [22] or the Grb2-binding sites (Fig. 4B, 4G) of FRS2α does not affect the self-renewal ability of NSCs. Although Erk activation, but not Akt activation, was moderately reduced in both the *Frs2α^4F^* (Fig. 4H, 4I) and *Frs2α^2F^* mutants [22], Erk activation via either the Shp2- or Grb2-binding sites of FRS2α appears to be sufficient for the self-renewal of NSCs. If both of these sites are lost, however, Erk activation may be severely diminished, and the self-renewal ability of NSCs may be compromised. To test this possibility, we blocked *Frs2α* translation in NSPCs with short hairpin (sh) RNA-expressing retroviral vectors. shRNAs for *Frs2α* (shFrs2α-1 or shFrs2α-2) effectively downregulated the expression of FRS2α (Fig. 5A). Then, we transduced the retroviral vectors into NSPCs from E14.5 telencephalons and cultured them in the presence of FGF2. We found that cells expressing shFrs2α-1 or shFrs2α-2 formed smaller and fewer secondary neurospheres than cells expressing control shRNA (Fig. 5B, 5C). Furthermore, neurospheres with decreased *Frs2α* expression showed strongly reduced activation of Erk in response to FGF2 stimulation, but the activation of Akt was not affected (Fig. 5D, 5E). These results suggest that the FRS2α-Erk axis, but not the FRS2α-Akt axis, contributes to the self-renewal of NSCs and to the efficient proliferation of NSPCs in response to FGF2.

To examine the relationship between the Erk activation level and self-renewal as well as the proliferation of NSPCs, we treated NSPCs from E14.5 mouse telencephalons with different doses of a MEK inhibitor U0126. We found that Erk activation was decreased in a dose-dependent manner (Fig. 5F). Next, we cultured them in the presence of FGF2. The resulting primary neurospheres were dissociated and cell numbers were counted; then, the same number of NSPCs were cultured again to form secondary neurospheres. Our results demonstrated that the number of cells and the number of neurospheres both were decreased with treatment with U0126 in a dose-dependent manner (Fig. 5G). It thus appears that there is a close correlation between the activation levels of Erk and the self-renewal of NSCs as well as the proliferation of NSPCs. Moreover, we noticed that the number of cells was more severely suppressed than that of neurospheres at the same dose of U0126. When we used U0126 at lower doses, less than 0.7 μM, the number of cells was moderately suppressed, while the number of neurospheres was not significantly changed (Fig. 5H).

To further examine the correlation between Erk activation, self-renewal, and proliferation, we used PD98058, another MEK inhibitor. PD98059 moderately inhibited Erk activation in a dose-dependent manner, but it was less effective than U0126 (Supporting Information Fig. 3A and Fig. 5F). Next, we treated NSPCs from E14.5 mouse telencephalons with different doses of PD98059 and cultured them in the presence of FGF2. The resulting primary neurospheres were dissociated and cell numbers were counted; then, the same numbers of NSPCs were cultured again to form secondary neurospheres. As a result, although the numbers of cells were decreased in a dose-dependent manner, there was little change in the number of neurospheres at any dose (Supporting Information Fig. 3B).

It appears that cell proliferation was inhibited in correlation with the moderately reduced levels of Erk activity; however, the inhibition levels of Erk activation by U0126 at low doses or by PD98059 were not sufficient for the inhibition of self-renewing activity in this assay condition.

Therefore, a low level of Erk activation appears to be sufficient for the self-renewal of NSCs, whereas a high level of Erk activation may be required for the full proliferative activity of NSPCs.

### Hes1 Is a Target of FGF2-FRS2α Signaling to Maintain NSPCs in an Undifferentiated State In Vitro

The identities of the targets of the FGF-FRS2α signaling pathway for the self-renewal of NSCs remain unknown. It is known that the transcription factor Bmi-1 [35] and Notch signaling [36] play an important role in the self-renewal of NSCs. Hes family proteins are transcription factors downstream of Notch signaling [36]. We next examined whether certain components of the Notch signaling pathway or Bmi-1 serve as targets of FGF signaling for the self-renewal of NSCs. Quantitative real-time reverse transcription polymerase chain reaction showed that the expression of *Bmi-1*, *Notch1*, *Notch2*, *Hes3*, or *Hes5* mRNA was not upregulated in NSPCs in response to FGF2 stimulation (Supporting Information Fig. 2). Interestingly, we found that the expression of *Hes1* was elevated and sustained for 24 hours in response to FGF2 in NSPCs (control; Fig. 5I).

To examine whether FRS2α is involved in FGF2-induced Hes1 expression, we knocked down the expression of *Frs2α* in NSPCs. We found that expression of *Hes1* mRNA was rapidly decreased to basal levels after 6 hours (Fig. 5I). Expression of Hes1 protein was not induced at significant levels by FGF2-stimulation (Fig. 5J). We next examined whether activation of Notch1 and Notch2 contributes to the FGF2-induced Hes1 expression. We used γ-secretase inhibitor DAPT, which blocks cleavage of the cytoplasmic domain of Notch and thereby inhibits Notch signaling. We found that treatment with U0126, but not DAPT, inhibited the FGF2-induced expression of Hes1 (Fig. 5K). FGF2 stimulation did not induce activation of Notch as shown by expression of the intracellular domain of Notch1 and Notch2, nor induce expression of Delta-like one (Dll1), a Notch ligand. Treatment with U0126 did not affect Notch activity (Fig. 5K). These results suggest that FGF2-induced expression of Hes1 is mediated by FRS2α, and is independent of Notch signaling but dependent on the activation of Erk.

To further examine whether the FGF2-FRS2α-Erk-Hes1 axis contributes to the self-renewal of NSCs, we expressed Hes1 in cultured NSPCs, in which *Frs2α* was knocked down or not, and examined the resulting number and size of the FGF2-induced neurospheres. Expression of Hes1 increased the number of FGF2-induced neurospheres in which FRS2α was intact (Fig. 5L). Moreover, although the knocking down of *Frs2α* reduced the number of FGF-induced neurospheres, the exogenous expression of Hes1 rescued this phenotype to some extent (Fig. 5L). In contrast, the exogenous expression of Hes1 neither significantly increase the size of neurospheres in which FRS2α was intact (data not shown) nor rescue the decreased size of the neurospheres caused by knocking down of *Frs2α* (Fig. 5B and data not shown). These results suggest that the FGF2-FRS2α-Hes1 axis promotes the self-renewal of NSCs.

### Hes1 Is a Target of FGF2-FRS2α Signaling to Maintain NSPCs in an Undifferentiated State In Vivo

**Figure 6:**
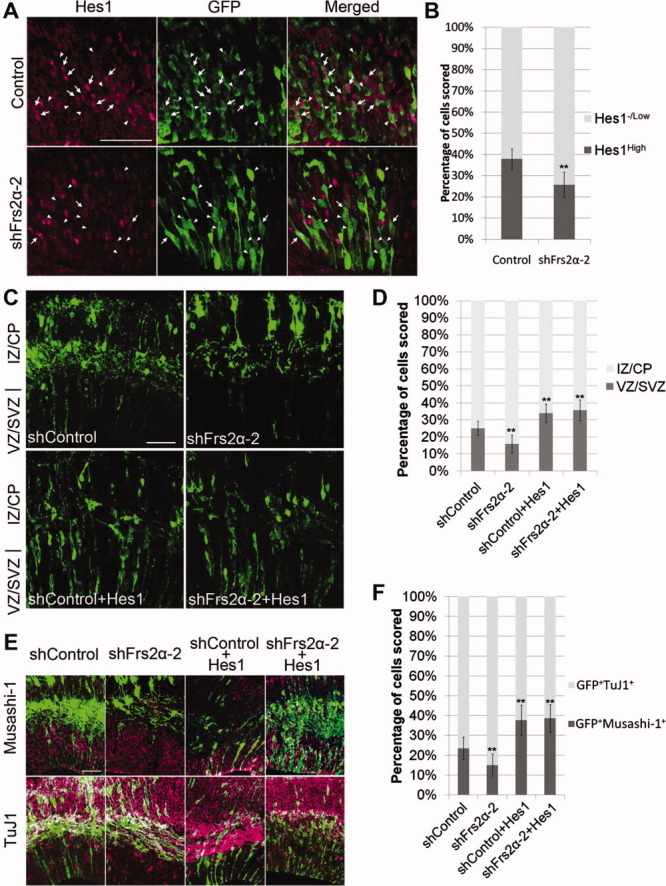
Hes1 is a target of fibroblast growth factor (FGF)-FGF receptor substrate 2α signaling for the self-renewal of neural stem cells. **(A):** Vectors expressing EGFP and shRNAs were coinjected into the lateral ventricles of E12.5 mouse embryos in utero and electroporated. Then, embryos were sacrificed at E13.5 and immunostained against Hes1 (magenta) and GFP (green). **(B):** GFP-positive cells expressing high (arrow) or low/negligible (arrowhead) levels of Hes1 were counted and the results are summarized. In each condition, more than 148 cells were counted in at least five randomly selected areas. Scale bars = 50 μm. **(C–F):** Vectors for the expression of GFP, Hes1, or shRNAs were coinjected into the lateral ventricles of E12.5 mouse embryos in utero and electroporated. Then, the embryos were sacrificed at E15.5, and cortical sections were immunostained against GFP (green) **(C)**, GFP and Musashi-1 (magenta) (**[E]**, upper panel), or GFP and TuJ1 (magenta) (**[E]**, lower panel). **(D, F):** Quantification of the data in **(C)** and **(E)**, respectively. In each condition, at least 400 cells were counted in 8–18 randomly selected areas. Abbreviations: CP, cortical plate; EGFP, Enhanced Green Fluorescent Protein; GFP, Green Fluorescent Protein; IZ, intermediate zone; SVZ, subventricular zone; VZ, ventricular zone.

To examine whether Hes1 is a target of signaling via FRS2α for the self-renewal of NSCs in vivo, we inhibited the expression of FRS2α in the developing cortex by shRNA for *Frs2α*. We coinjected expression vectors for GFP and an shRNA for *Frs2α* or control into the telencephalic lateral ventricles of E12.5 cortex and performed in utero electroporation. Then, embryos were sacrificed at E13.5 and immunostained with antibodies against Hes1 and GFP. We found that knocking down of *Frs2α* caused a decrease in expression levels of Hes1 in the cortex (Fig. 6A, 6B). We next coinjected those expression vectors with or without vectors expressing *Hes1*. The knocking down of *Frs2α* reduced the number of cells in VZ/SVZ (Fig. 6C, 6D). When exogenous *Hes1* was coexpressed, many control or *Frs2α* knocked-down cells were localized in the VZ/SVZ region (Fig. 6C, 6D). Hence, the exogenous expression of Hes1 increased the number of NSPCs and rescued the reduced NSPCs caused by the knocking down of *Frs2α in vivo.* Double-immunostaining with antibodies against GFP and TuJ1 or Musashi-1 showed that knocking down of *Frs2α* increased the ratio of neurons at the expense of NSPCs (Fig. 6E and 6F). On the other hand, when exogenous Hes1 was coexpressed, many control or *Frs2α* knocked-down cells were Musashi-1-positive, suggesting that they remained undifferentiated (Fig. 6E and 6F). However, exogenous Hes1 expression did not increase the number of mitotic cells that were positive for pH3 in the VZ/SVZ region (Supporting Information Fig. 4). These results suggest that the FRS2α-Hes1 axis maintains undifferentiated NSPCs in vivo; however, exogenous Hes1 expression does not significantly promote cell division.

**Figure 7:**
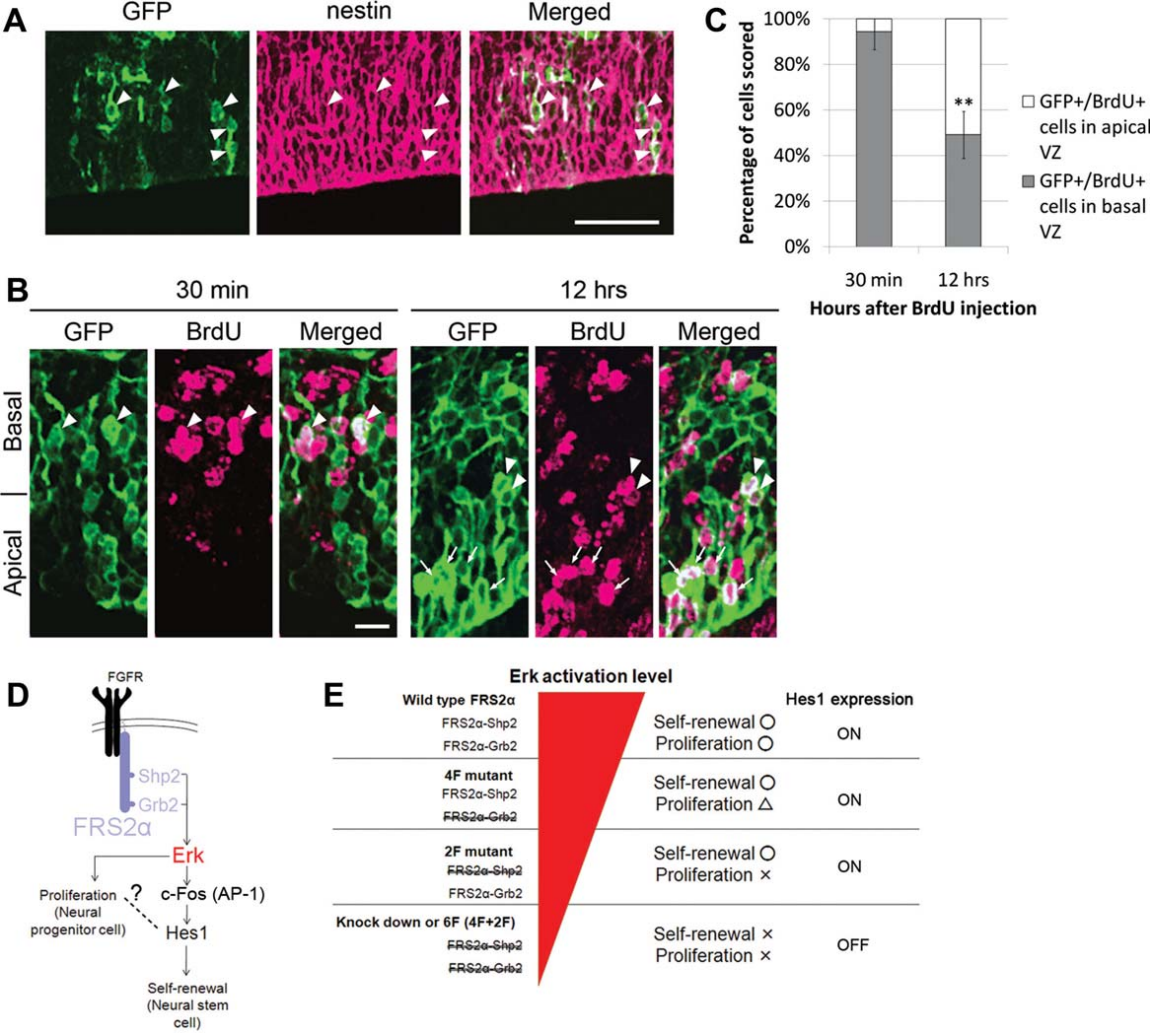
Cells expressing Hes1 show radial glial properties **(A–C)**. **(A, B):** Vectors expressing Hes1, GFP, and shRNA for FRS2α were coinjected into the lateral ventricles of E13.5 mouse embryos in utero and electroporated. Then, the embryos were sacrificed at E15.5, and cortical sections were immunostained against GFP (green) and nestin (magenta; **[A])** or BrdU (magenta; **[B]**). BrdU was administered 30 minutes or 12 hours before sacrifice. **(A):** Most GFP-positive cells expressed nestin, as indicated by arrowheads. Scale bar = 50 μm. **(B):** The cell bodies of most GFP-positive cells in the S-phase (30 minutes after BrdU administration) were located at the basal half of VZ (arrowhead). In contrast, the cell bodies of GFP-positive cells in the G1 phase were present at both the basal and apical (arrow) half of VZ. Scale bar = 10 μm. **(C):** Quantification of the data in **(B)**. In each condition, at least 145 cells were counted in 19–29 randomly selected areas. **(D):** Fibroblast growth factor (FGF)-induced Erk activation via Shp2- or Grb2-binding sites of FRS2α contributes to both proliferation of neural stem/progenitor cells (NSPCs) and self-renewal of neural stem cells (NSCs). The latter is at least partly mediated by Hes1, whose expression may be induced by the binding of AP-1 complex to the Hes1 promoter. **(E):** For the normal proliferation of NSPCs in response to FGF, strong Erk activation via both Shp2- and Grb2-binding sites of FRS2α is required. On the other hand, relatively weak Erk activation levels are sufficient to activate the self-renewal switch of NSCs with Hes1 expression, the master regulator for stemness. Abbreviations: BrdU, Bromodeoxyuridine; FGFR, Fibroblast growth factor receptor; FRS2α, FGF receptor substrate 2α; GFP, Green Fluorescent Protein; VZ, ventricular zone.

To confirm that the rescued cells are bona fide NSCs, we examined whether they express nestin and undergo INM in a similar way analyzed for Figure 3. Most cells in VZ expressing Hes1 also expressed nestin (97.3% ± 3.5%, total 518 cells counted in 18 randomly selected areas; Fig. 7A). Although the GFP-positive cell bodies in the S-phase were present at the basal side of VZ, many of them were located at the apical side in the G1 phase (Fig. 7B and 7C). Thus, these cells appear to be bona fide NSCs. It thus appears that Hes1 is a downstream target of FGF-FRS2α signaling for the self-renewal of NSCs in vivo.

As NSCs derived from the *Frs2α^2F^* mutant appear to have intact self-renewing activity [22], the expression of Hes1 should be intact in NSPCs in these mutant mice. We found that full levels of Hes1 expression were induced by FGF2 stimulation in *Frs2α^2F^* NSPCs in vitro (Supporting Information Fig. 5A). We also found that there was little difference in the expression levels of Hes1 in the cortex between the wild-type/heterozygote and the mutant (Supporting Information Fig. 5B). In addition, we examined expression levels of Hes1 in *Frs2α^4F^* mutant cortex. As expected, we found little difference in expression levels of Hes1 between wild-type/heterozygote and the *Frs2α^4F^* mutant (data not shown). Moreover, though treatment with PD98059 moderately reduced FGF2 induced-Erk activation, it did not affect Hes1 expression (Supporting Information Fig. 5C). These results further support the notion that Hes1 is a low level target of Erk activated by FGF-FRS2α signaling.

To examine the molecular mechanisms of Hes1 induction by FGF-FRS2α signaling, we performed a luciferase assay with a reporter vector containing the promoter region of *Hes1* (–2570 to +277) in cultured NSPCs. FGF2 stimulation increased promoter activity, and this was inhibited by treatment with U0126 (Supporting Information Fig. 6A). Serial deletion analysis of the promoter (−750, −487, and −12) showed that FGF2 stimulation increased the promoter activity up to −750, but this was inhibited in −487 or −12 construct (Supporting Information Fig. 6B). In the promoter region between −750 to −487, we identified a potential binding sequence of AP-1 (−498 to −492, TGACTCC) [37], an important transcriptional activator downstream of Erk, composed of members of Jun and Fos families [38]. Point mutations in this sequence (TGACTCC to CGTCTAC) abolished the activation of the reporter by FGF2 stimulation (Supporting Information Fig. 6B). The expression of *c-Fos*, a member of the Fos family, was rapidly induced after FGF2 stimulation in NSPCs (Supporting Information Fig. 6C), and chromatin immunoprecipitation assay showed that c-Fos interacted with the promoter region containing the putative AP-1-binding site in response to FGF2 stimulation (Supporting Information Fig. 6D). FGF2 stimulation transiently induced expression of c-Fos, reaching peak levels at ∼80 minutes, and formation of AP-1 complex, and then expression of c-Fos gradually declined to basal levels. On the other hand, expression levels of c-Jun were stable (Supporting Information Fig. 6E). Moreover, knocking down of *c-Fos* decreased FGF2-induced Hes1 expression (Supporting Information Fig. 6F). These results suggest that Erk activation by FGF2 stimulation induces the expression of *c-Fos*, and then, AP-1 containing c-Fos binds to the *Hes1* promoter and activates transcription.

It has been reported that expression of Hes1 oscillates in neural progenitor cells, though precise molecular mechanisms are still unclear. To examine whether FGF signaling is involved in this process, we incubated cultured NSPCs in the starvation media without growth factors for overnight, stimulated them with FGF2, and monitored the expression levels of Hes1, c-Fos, and c-Jun at every 20 minutes (Supporting Information Fig. 6G). After starvation, expression of Hes1 was at very low levels. Upon stimulation with FGF2, expression of Hes1 was strongly induced, reaching a peak level after ∼100 minutes and declined to a basal level after ∼180 minutes. Then Hes1 expression was increased again and reached the second peak level after ∼240 minutes, though the expression levels at the second peak were lower than those of the first. Expression of c-Fos was strongly induced by FGF2 stimulation, then declined gradually to a basal level and did not show the second peak (Supporting Information Fig. 6E and data not shown). When *c-Fos* was knocked down, expression levels of Hes1 was decreased without showing the second peak (data not shown). We also monitored phosphorylation levels of Erk in the same time course and found that levels of phosphorylation of Erk was transiently induced as those of c-Fos without showing the second peak, while expression levels of Erk were stable (Supporting Information Fig. 6E and data not shown). These results suggest that the FGF-AP-1 signaling is important for the induction of Hes1 expression to initiate oscillation in cultured NSPCs.

## DISCUSSION

In this report, we dissected the role of FGF signaling for the self-renewal and proliferation of NSPCs. First, FRS2α, a central mediator of FGF signaling, appears to selectively activate Erk and induces little activation of Akt. Then, it is likely that low levels of Erk activation is sufficient for induction of Hes1, which allows NSCs to self-renew. Further, high levels of Erk activation may be required for full levels of proliferation of NSPCs (Fig. 7D and 7E).

### FRS2α Controls the FGF-Erk Axis by the Degree of Tyrosine Phosphorylation on Grb2- and Shp2-Binding Sites

This notion is well-supported by the phenotype of *Frs2α* mutants and NSPCs expressing reduced levels of FRS2α by the expression of shRNA for *Frs2α*. The *Frs2α* knocked-down NSPCs showed impaired self-renewing activity with reduced Hes1 expression and impaired proliferation. The Shp2-binding site mutant *Frs2α^2F^-*derived NSPCs showed intact self-renewing activity with intact Hes1 expression but impaired proliferation. The Grb2-binding site mutant *Frs2α^4F^-*derived NSPCs showed intact self-renewing activity but slightly impaired proliferation. On the other hand, FGF2-induced activation of Erk was most impaired in the *Frs2α* knocked-down NSPCs among the wild-type, *Frs2α^2F^,* and *Frs2α^4F^* NSPCs. We previously reported that the *Frs2α^4F^* mutant is able to activate Erk at slightly higher levels than the *Frs2α^2F^* mutant, although both mutants show reduced levels of Erk activation than wild-type FRS2α [13]. Therefore, it is likely that the differences in the activities of self-renewal and proliferation of NSPCs in each FRS2α mutant is at least partly due to different levels of Erk activity.

In other words, we demonstrated that FRS2α controls the FGF-Erk axis by the degree of tyrosine phosphorylation on Grb2- and Shp2-binding sites (Fig. 7D and 7E). Low Erk activation levels via at least one of two tyrosine phosphorylation sites of FRS2α are sufficient for FGF-dependent enhancement of Hes1 expression and self-renewal of NSCs. In contrast, strong Erk activation via both the tyrosine phosphorylation sites are required for the full proliferation of NSPCs.

Consistent with our results, there is a convincing evidence that Erk2 is required for the full activity of self-renewal of NSCs and proliferation of NSPCs in response to FGF2, based on the studies using conditional Erk2 knockout mice [39]. It was also reported that adhesion signals by β1 integrin partly contributes to Erk activation for NSC maintenance [40]. Coordinated signaling of both growth factors such as FGF and adhesion such as β1 integrin would be essential for NSC maintenance.

### FGF-FRS2α-Erk Signaling Regulates Hes1 Expression and Self-Renewal of NSCs

It is known that Hes1 plays important roles for self-renewal of NSCs. Hes1 knockout mice show defects in self-renewal of NSCs [28]. In this article, the authors also showed that proliferation of NSPCs is not significantly affected in the Hes1 knockout mice. These findings are consistent with our results.

Because it has been reported that the expression level of *Hes5*, but not *Hes1*, is reduced in *Notch1* or *CBF1* mutant mice, the expression of *Hes1* has been predicted to also be regulated by signaling cascades other than Notch signaling [36]. Our results suggest that FGF-FRS2α-Erk signaling is indeed a distinct pathway from Notch signaling for the self-renewal of NSCs. This notion is supported by the result that knockdown of *Frs2α* led to reduced self-renewing activity of NSCs, and the coexpression of Hes1 rescued the phenotype in vitro and in vivo. Consistent with this, it was reported that overexpression of Hes1 or activated forms of either Notch1 or FGF receptor two increases the self-renewing activity of NSCs in the cortex [23,41,42].

We showed that FGF activates *Hes1* promoter dependent on activation of Erk and our results suggest that the transcription factor complex AP-1 binds to the AP-1 consensus binding site of *Hes1* promoter. Therefore, it appears that FGF-Erk axis induces expression of *Hes1* at the transcriptional levels.

It is known that Hes1 expression oscillates in many cell types, including presomitic mesoderm and many cultured cells [43]. During mouse somitogenesis, oscillation of Hes7, another Hes family member, is initiated by FGF signaling and propagated or maintained by Notch signaling [44]. It was reported that the oscillation of Notch-induced Hes1 expression regulates the maintenance of NSCs [45]. Accumulating evidence indicates that negative feedback mechanisms lead to oscillatory responses [46]. Hes1 serves as a transcriptional repressor, acting in an autoregulatory loop in the negative feedback mechanisms. Hes1 represses its own promoter, resulting in disappearance of *Hes1* mRNA and Hes1 protein. Then the disappearance of Hes1 protein leads to relief from the repression and allows the next round of expression. It was recently reported that FGF induces Hes1 expression in an oscillatory manner in a mesenchymal C3H 10T1/2 cell line [47]. In these cells, Erk activation also oscillates due to negative feedback mechanisms through Sos protein, a Ras activator. As we did not observe oscillation of FGF2-induced c-Fos expression or Erk activation, the autoregulatory loop involving Hes1 and Notch signaling might be sufficient for the Hes1 oscillation in NSPCs. Together with our results it appears that FGF-FRS2α-Erk-AP-1 signaling is important for the induction of Hes1 expression to the levels at which oscillation takes place in NSPCs. It is possible that FGF signaling keeps basal levels of *Hes1* transcription. Nevertheless, it is possible that FGF signaling contributes to regulation of oscillation at another level. We found that the second peak levels of Hes1 expression in FGF2-stimulated NSPCs were greatly reduced compared with the first peak levels. This may be because cells were desynchronized in the second round of Hes1 expression as previously described in other cells [43]. Therefore, to examine the precise role of FGF signaling for Hes1 oscillation in NSPCs, it would be important to monitor cycling activity of *Hes1* promoter in individual cells by real-time imaging as previously reported [42,43].

## CONCLUSION

In response to FGF2, FRS2α signals increase Erk phosphorylation levels, controlled by the degree of its tyrosine phosphorylation. This leads to qualitatively different biological outputs: self-renewal at least partly mediated by Hes1, the master regulator of stemness, versus cell proliferation. The molecular mechanisms by which FRS2α controls NSCs could be generalized to a variety of other stem cells. Certain methods for modifying the functions of FRS2α may be useful for obtaining in vitro cultures of stem cells of desired quality.

## Disclosure of Potential Conflicts of Interest

The authors indicate no potential conflicts of interest.
