# Supplementary figures and images for: FRS2α Regulates Erk Levels to Control a Self-Renewal Target Hes1 and Proliferation of FGF-Responsive Neural Stem/Progenitor Cells
Source: Stem Cells. 2010 Jul 22;28(9):1661–73. doi: 10.1002/stem.488 (PMC2996081; doi:10.1002/stem.488)

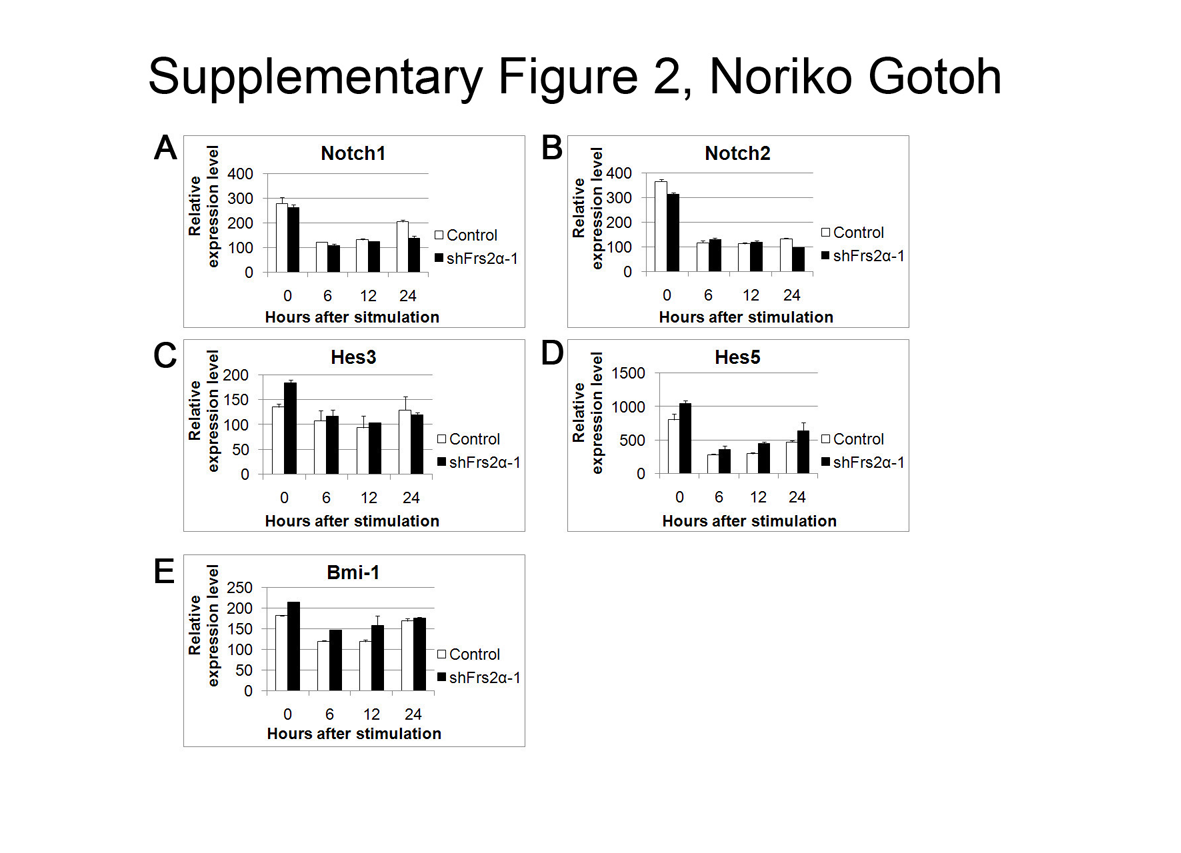

Supplement: Supplementary file 1 [file stem0028-1661-SD1.tif]

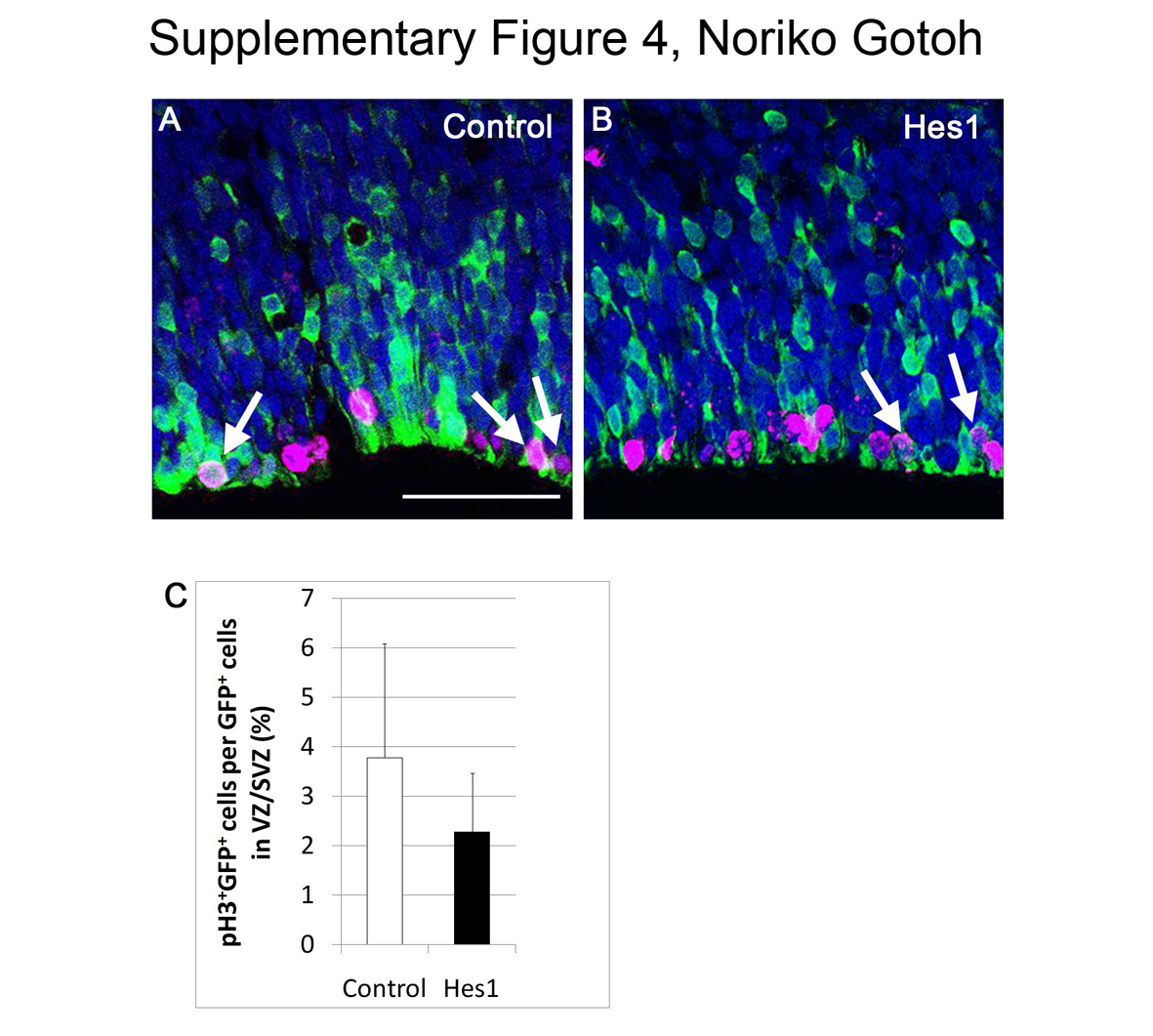

Supplement: Supplementary file 2 [file stem0028-1661-SD2.tif]
