# Supplementary material for: FRS2α Regulates Erk Levels to Control a Self-Renewal Target Hes1 and Proliferation of FGF-Responsive Neural Stem/Progenitor Cells
Source: Stem Cells. 2010 Jul 22;28(9):1661–73. doi: 10.1002/stem.488 (PMC2996081; doi:10.1002/stem.488)
Supplement: Supplementary file 3 [file stem0028-1661-SD3.doc]

**Supplementary Figure Legends**

**Supplementary Figure 1.** (**A, B**) Overexpression of FRS2α *in vitro*. The NSPCs obtained from the telencephalons of E12.5 mouse embryos were transduced with retroviral expression vectors and cultured in the presence of 2, 5, or 20 ng/ml of FGF2. Expression of FRS2α or FRS2α-8V significantly increased the size (**A**) and number (**B**) of tertiary neurospheres at every dose of FGF2, but not 6F.

**Supplementary Figure 2.** Quantitative RT-PCR analysis. **(A–E)** Adherent cultured NSPCs infected with the virus were stimulated with 20 ng/ml of FGF2. Expression levels of *Notch1* (**A**), *Notch2* (**B**), *Hes3* (**C**), *Hes5* (**D**), and *Bmi-1* (**E**) were not upregulated in response to FGF2 stimulation.

**Supplementary Figure 3.** (**A**) NSPCs from E14.5 telencephalons were cultured in the presence of FGF2 together with different doses of PD98059. Then, the cells were lysed 6 h or 10 h after the initiation of culture. The signal intensities of pErk1/2 and Erk1/2 were quantified, and represented as the relative activity of pErk against Erk1/2 (right panel). (**B**) NSPCs were cultured in the presence of FGF2 together with different doses of PD98059 to form neurospheres. The resulting primary neurospheres were dissociated to single cells and the cell number was counted. Then, the same numbers of NSPCs were cultured again to form secondary neurospheres, and the number of resulting neurospheres was determined.

**Supplementary Figure 4.** Exogenous expression of Hes1 did not promote cell division. Vectors expressing Hes1 and GFP (**B**) or GFP alone (**A**) as a control were electroporated into the cortex of E12.5 mouse embryos, and the embryos were fixed at E13.5. The sections were immunostained against GFP (green) and pH3 (magenta), and the ratio of double-positive cells (arrow) to GFP-positive cells in VZ/SVZ was determined by counting 4–5 randomly selected areas for each condition (**C**). The ratios were not significantly different. Nuclei were stained in blue. Scale bar, 50 μm. Error bars, S.D.

**Supplementary Figure 5.** Hes1 expression was normal in *Frs2α^2F/2F^* NSPCs. (**A**) NSPCs from E14.5 telencephalons were adherently cultured in the presence of EGF. Next, NSPCs were starved for 15 h in the absence of growth factors and then stimulated with 20 ng/ml FGF2. Lysates were prepared at each time point and subjected to western blotting with anti-Hes1 or Erk1/2 antibodies. (**B**) Expression levels of Hes1 in E14.5 cortex of wild-type/heterozygous (+/H) or homozygous embryos were examined by immunohistochemistry with anti-Hes1 antibody. Hes1 expression levels were not decreased in *Frs2α^2F^* NSPCs or in the cortex. (**C**) NSPCs were starved and pretreated with DMSO or 25 μM PD98059 for 1 h before stimulation with FGF2. Then, cells were lysed at 2 h after the stimulation for immunoblotting (left panel), and relative signal levels of Hes1 expression or Erk1/2 phosphorylation against Erk1/2 was quantified (right panel).

**Supplementary Figure 6.** Analysis of *Hes1* promoter**.** (**A**) NSPCs from E14.5 telencephalons were transfected with *Hes1* promoter (–2570 to +277), and pretreated for 1 h with DMSO (vehicle) or 25 μM U0126 before FGF2 stimulation, and subjected to luciferase assay. (**B**) NSPCs were transfected with reporter vectors with serial deletions (–2570, –750, –487, and –12 to +277) or its mutant (–750_m). Then, the cells were stimulated with FGF2 and subjected to luciferase assay. (**C**) Neurospheres were stimulated with FGF2, and RNA was extracted at the time points indicated. Then, the expression levels of *c-Fos* were quantified by real-time qRT-PCR. (**D**) NSPCs were stimulated with FGF2 for 1 h, and chromatin was immunoprecipitated with normal rabbit IgG (control) or anti-c-Fos antibodies. Then, PCR was performed using primers targeting a putative AP-1-binding site (AP-1) or another region (NC) of the Hes1 promoter (left panel). Real-time PCR was also performed to quantify enrichment of putative AP-1 binding site (right panel). (E) NSPCs were stimulated with FGF2, and lysed at indicated time points. The lysates were subjected to immunoblotting (Input), or immunoprecipitation with anti-c-Fos antibody (IP) followed by immunoblotting. Relative signal levels of c-Fos expression against c-Jun in the lysates was quantified (lower panel). (F) NSPCs transfected with siRNAs were starved and stimulated with FGF2. The cells were lysed at 100 min after the stimulation, and the lysates were subjected to immunoblotting. (G) NSPCs were stimulated with FGF2, and lysed at indicated time points. The lysates were subjected to immunoblotting (upper panel), and relative signal levels of Hes1 expression against c-Jun was quantified (lower panel). We repeated the experiments three times and obtained similar results. Representative data were shown.

**Supplementary Materials and Methods**

**Animals.**

Experiments with animals were carried out in accordance with the guidelines for animal use issued by the Committee of Animal Experiments, Institute of Medical Science, University of Tokyo. Plug dates were deemed E0.5. Wild-type ICR mice were purchased from Japan SLC (Shizuoka, Japan, http://www.jslc.co.jp). Generation of *Frs2α^4F^* and *Frs2α^2F^* mutant mice and genotyping methods were described previously [15].

**Vectors.**

For the expression of FRS2α or Hes1, the coding sequence of mouse wild-type FRS2α, Hes1 or the mutants FRS2α-6F or FRS2α-8V was subcloned into the multiple cloning sites of a pMYs-IG retroviral vector [1] with BamHI and NotI sites (FRS2α or the mutants) or BamHI and EcoRI sites (Hes1). The sequence of 6F and 8V was reported previously [2-3]. The pMYs-IG vector contains sequences for an internal ribosomal entry site (IRES) and enhanced green fluorescent protein (EGFP) downstream of the inserted coding sequence, and viral infections were monitored based on the expression of the EGFP translated from the IRES. For the expression of shRNAs by retroviral vectors, we used pSIREN-retroQ (containing a puromycin resistance gene) (Clontech), pSIREN-ZsGreen (including the reporter gene *ZsGreen*) (Clontech) or pSSCG. To construct the pSSCG vectors, target sequences were first sub-cloned to pSK-mU6 vector containing the mouse U6 promoter. Then, the promoter-shRNA cassette was sub-cloned into pSSCG vector, which expresses EGFP under the control of CMV promoter. For the expression of shRNAs by lentiviral vectors, target sequences were first sub-cloned to pENTR4-H1 containing the human H1 promoter. Then, the LR recombination reaction was performed between the entry clones and the pCS-RfA-EG lentiviral vector, which has EF-1α promoter and EGFP sequences to monitor viral infection, using Gateway LR Clonase (Invitrogen) according to the manufacturer’s instructions. The target sequences used to block *Frs2α* translation were 5’-GAGAAGACCTGCACTATTA-3’ (shFrs2α-1) and 5’-GCATAACTATGTTAATACAGA-3’ (shFrs2α-2). The control sequences were 5’-GAAGGCCAGACGCGAATTATT-3’ (targeting *LacZ*, Figure 7), 5’-GAGAGGACCTGTACTACTA-3’ (mutated form of shFrs2α-1, Figure 5D and E, Figure 6A and B, and Supplementary Figure 3), and 5’-GTGCGTTGCTAGTACCAAC-3’ (targeting *luciferase*, Figure 5A-C). EGFP and Hes1 were expressed with pCXN2 and pCAGGS vectors, respectively, which are driven by the CAG promoter.

**Western blotting.**

For the stimulation of NSPCs, the cells were starved for 14 to 15 hours in basal medium without growth factors and then stimulated with 20 ng/ml FGF2. The cells or tissue lysates were subjected to immunoblotting with rabbit polyclonal anti-p44/42 MAP kinase (1:1000; Cell Signaling), rabbit polyclonal anti-phospho-p44/42 MAP kinase (1:1000; Cell Signaling), rabbit polyclonal anti-Akt (1:1000; Cell Signaling), rabbit polyclonal anti-phospho-Akt (Ser473) (1:1000; Cell Signaling), rabbit polyclonal anti-Hes1 [4] (1:2000), rabbit polyclonal anti-cleaved Notch1 (1:1000; Abcam), rabbit polyclonal anti-cleaved Notch2 (1:1000; Abcam), mouse monoclonal anti-Notch1 (1:1000; Santa Cruz Biotechnology), rabbit polyclonal anti-Notch2 (1:1000; Santa Cruz Biotechnology), rabbit polyclonal anti-Dll1 (1:1000; Santa Cruz Biotechnology), rabbit polyclonal anti-c-Fos (1:1000; Santa Cruz Biotechnology), rabbit polyclonal anti-c-Jun (1:1000; Santa Cruz Biotechnology), rabbit polyclonal anti-SH-PTP2 (C18) (1:2000; Santa Cruz Biotechnology), rabbit polyclonal anti-phospho-SHP2 (Tyr542) (1:1000; Cell Signaling), rabbit polyclonal anti-Gab1 (1:1000; Cell Signaling), rabbit polyclonal anti-phospho-Gab1 (1:1000; Cell Signaling), mouse monoclonal anti-actin (1:5000; Chemicon), rabbit polyclonal phospho-FRS2α (1:1000; Cell Signaling), or rabbit polyclonal anti-FRS2α (1:1000; produced in our laboratory, see below) antibodies. Rabbit-polyclonal anti-FRS2α antibody was generated against a glutathione-S-transferase fusion protein containing the C-terminal portion of FRS2α (amino acids 400-508). The rabbit serum was subjected to affinity purification. The secondary antibodies used were horseradish peroxidase (HRP)-conjugated anti-mouse IgG (1:10,000; Amersham) or HRP-conjugated anti-rabbit IgG (1:5000; Amersham).

**In utero electroporation.**

Gene transfer into ICR mouse brains by *in utero* electroporation was performed using a Nepagene CUY21EDIT electroporator. One microliter of plasmid solution dissolved in phosphate buffered saline (PBS) was injected into the lateral telencephalic ventricle with a pulled capillary micropipette controlled by a FemtoJet microinjector (Eppendorf). The concentrations of the plasmids were 1 μg/μl for the pCXN2-EGFP or 2 μg/μl for the other vectors. Electric pulses were applied at 30 V for 50 ms, followed by a 950 ms gap; this process was repeated five times while the uterus was kept wet with PBS. A forceps-type electrode (CUY650P3, 3 mm diameter platinum round plates, Nepagene) was used for pulse delivery.

**Immunohistochemistry.**

The antibodies used for immunohistochemistry were rabbit polyclonal anti-GFP (1:500, MBL), mouse monoclonal anti-Nestin (1:500, BD), rat monoclonal anti-BrdU (1:250, Abcam), mouse monoclonal anti-βIII-tubulin (TuJ1) (1:500, Covance), rabbit polyclonal anti-phospho-histone H3 (Ser10) (1:500, Upstate), Rat anti-Musashi-1 [5] (1:400), guinea pig anti-Hes1 [6-7] (1:500), Alexa488-conjugated anti-rabbit IgG (1:500, Molecular Probes), Biotin-conjugated anti-guinea pig IgG (1:200, Jackson Immuno Research), Alexa546-conjugated streptavidin (1:1000, Molecular Probes), Alexa546-conjugated anti-rat IgG (1:500, Molecular Probes), and Alexa546-conjugated anti-mouse IgG (1:500, Molecular Probes). For BrdU labeling, mice were injected intraperitoneally with 200 mg/kg BrdU.

**qRT-PCR.**

Adherent cultured NSPCs were starved for 14 hours in basal medium without growth factors and insulin and then stimulated with 20 ng/ml FGF2. Total RNA was prepared using the RNeasy kit (Qiagen) and reverse transcribed into complementary DNA with Primescript (TaKaRa Bio). qRT-PCR was performed with ABI StepOne^TM^ real-time PCR system and SYBR premix ExTaq (TaKaRa Bio). The primer sequences used were 5’-TGACAGGATGCAGAAGGAGA-3’ (*β-actin*, forward), 5’-GCTGGAAGGTGGACAGTGAG-3’ (*β-actin*, reverse), 5’-CCACTTAATGTGTGTCCTGTGTG-3’ (*Bmi-1*, forward), 5’-AATAGGACAATACTTGCTGGTCTCC-5’ (*Bmi-1*, reverse), 5’-GGTGAAGACCGTGTCAGGAG-3’ (c-Fos, forward), 5’-TCCGATTCCGGCACTTG-3’ (c-Fos, reverse), 5’-CCGCAAGAGGCTTGAGATG-3’ (*Notch1*, forward), 5’-CTGCAGACACAGGCTTCAGTG-3’ (*Notch1*, reverse), 5’-GCTGACTGCACAGAAGACGTG-3’ (*Notch2*, forward), 5’-GGAAGGCGCCATCTGTATTC-3’ (*Notch2*, reverse), 5’-ACTGTGAAGCACCTCCGGAAC-3’ (*Hes1*, forward), 5’-GGTCACCTCGTTCATGCACTC-3’ (*Hes1*, reverse), 5’-GCAGCTGAGGTCTCTTCTGGA-3’ (*Hes3*, forward), 5’-TGGCCAGAGTCCTTGCAGT-3’ (*Hes3*, reverse), 5’-AGCTGGAGAAGGCCGACATC-3’ (*Hes5*, forward), and 5’-TAGTCCTGGTGCAGGCTCTTG-3’ (*Hes5*, reverse). Expression of β-actin was used to normalize the samples.

**Luciferase assay.**

All Hes-1 promoter sequences (-2570, -750, -487, or -12 to +277) were sub-cloned into a pGL3 vector (Promega) encoding firefly luciferase. The PCR primers used to introduce mutations in the putative AP-1 binding site (-498 to -492) were 5’-CCTCATGAGCAGTTTCGTCTACCCTAAACATACAG-3’ (forward), 5’-CTGTATGTTTAGGGTAGACGAAACTGCTCATGAGG-3’ (reverse). Adherent cultured NSPCs from E14.5 telencephalon on 12-well plates were transfected with pGL3-Hes1 and pRL-TK *Renilla* control luciferase vector (Promega) using Lipofectamine 2000 (Invitrogen). Then, the cells were starved for 15 hours in basal medium lacking growth factors and stimulated with 20 ng/ml FGF2. The cells were lysed 36 hrs after FGF2 stimulation, and the extracts were analyzed using a Dual-Luciferase Reporter Assay System (Promega) and a luminometer Luminescencer-PSN (ATTO). The activity of the firefly luciferase was normalized by the *Renilla* luciferase activity, and represented as fold of the normalized activity of FGF-stimulated cells dividing by the activity from non-stimulated cells.

**ChIP assay.**

ChIP assay was preformed with the ChIP Assay Kit (MILLIPORE), according to the manufacturer’s protocol. Briefly, adherent cultured NSPCs from E14.5 telencephalon were starved for 15 hours in basal medium lacking growth factors and stimulated with 20 ng/ml FGF2. After 1 hour, the cells were fixed by formaldehyde (1 %, final concentration). Then, the cells were pelleted and lysed in the SDS Lysis Buffer (MILLIPORE). The lysates were subjected to sonication with a sonicator BIORUPTOR (COSMO BIO), to reduce the DNA length between 200 and 1000 base pairs. The lysates were then diluted with ChIP Dilution Buffer (MILLIPORE), and precleared with the Protein A Agarose/Salmon Sperm DNA (MILLIPORE). The supernatant was incubated with normal rabbit IgG (Santa Cruz Biotechnology) or anti-c-Fos (Santa Cruz Biotechnology) antibodies. Then, the immunocomplexes were collected with the Protein A Agarose/Salmon Sperm DNA and the DNA was eluted and reverse-crosslinked, followed by treatment with proteinase K. DNA was purified by phenol-chloroform-ethanol precipitation, and used as templates for PCR to amplify Hes1 promoter regions including putative AP-1 binding sites (-642 to -68; AP-1) or other region as a negative control (-2425 to -2092; NC). DNA before immunoprecipitation was used as an input. For PCR, the primer sequences used were 5’-AGAGAGGAATGAATGGGCTAGAG-3’ (AP-1, forward ), 5’-CCAAACTTTCTTTCCCACAGTAAC-3’ (AP-1, reverse), 5’-AAAGGCACTGTTAATGAACTACACC-3’ (NC, forward), 5’-CATGTGAAAGATTTCTCCAGCTC-3’ (NC, reverse). For real-time PCR, the primer sequences used were 5’-GGGCATGTTTAGCGTGTGG-3’ (forward), 5’-GGGAACAAGGTAAAGAGGATGTG-3’ (reverse).

**siRNA.**

We used siRNAs with following sequences; 5’-AUAAAGUUGGCACUAGAGACGGACA-3’ (c-Fos, sense), 5’-UGUCCGUCUCUAGUGCCAACUUUAU-3’ (c-Fos, antisense), 5’-AUAGGAUUGAACGGGAUCCAGAACA-3’ (control, sense), 5’-UGUUCUGGAUCCCGUUCAAUCCUAU-3’ (control, antisense). All siRNAs were purchased from Invitrogen. Lipofectamine RNAiMAX reagent (Invitrogen) was used to transfect siRNAs. Two days after the transfection, NSPCs were starved for 15 hrs, and then stimulated with 20 ng/ml FGF2. The cells were lysed at 100 min after the stimulation, and the lysates were subjected to immunoblotting.

**Reference**

1 Kitamura T, Koshino Y, Shibata F, et al. Retrovirus-mediated gene transfer and expression cloning: powerful tools in functional genomics. Exp Hematol***.*** 2003;31:1007-1014.

2 Hadari YR, Gotoh N, Kouhara H, et al. Critical role for the docking-protein FRS2 alpha in FGF receptor-mediated signal transduction pathways. Proc Natl Acad Sci U S A***.*** 2001;98:8578-8583.

3 Lax I, Wong A, Lamothe B, et al. The docking protein FRS2alpha controls a MAP kinase-mediated negative feedback mechanism for signaling by FGF receptors. Mol Cell***.*** 2002;10:709-719.

4 Ito T, Udaka N, Yazawa T, et al. Basic helix-loop-helix transcription factors regulate the neuroendocrine differentiation of fetal mouse pulmonary epithelium. Development***.*** 2000;127:3913-3921.

5 Kaneko Y, Sakakibara S, Imai T, et al. Musashi1: an evolutionally conserved marker for CNS progenitor cells including neural stem cells. Dev Neurosci***.*** 2000;22:139-153.

6 Shimojo H, Ohtsuka T, Kageyama R. Oscillations in notch signaling regulate maintenance of neural progenitors. Neuron***.*** 2008;58:52-64.

7 Baek JH, Hatakeyama J, Sakamoto S, et al. Persistent and high levels of Hes1 expression regulate boundary formation in the developing central nervous system. Development***.*** 2006;133:2467-2476.
